# Round 'Ere: Developing a novel method to engage community on the design of wellbeing data hubs

**DOI:** 10.23889/ijpds.v9i5.2831

**Published:** 2024-09-18

**Authors:** Dickman Gareta, Evelyn Lauren, Khumbo Shumba, Kobus Herbst, Dorina Onoya, Jacob Bor

**Affiliations:** 1 Africa Health Research Institute; 2 Department of Biostatistics, Boston University School of Public Health; 3 Health Economics and Epidemiology Research Office, Department of Internal Medicine, School of Clinical Medicine, Faculty of Health Sciences, University of the Witwatersrand; 4 DSI-SAMRC South African Population Research Infrastructure Network; 5 Department of Epidemiology, Boston University School of Public Health; 6 Department of Global Health, Boston University School of Public Health, Boston

The promise of community benefit from health data linked at scale is often missing the perspective of communities themselves. This paper outlines a recent project on developing a peer research and public engagement methodology to build community-designed linked and open wellbeing data hubs. Drawing on action research and appreciative inquiry, we aimed to develop a novel method to engage community on the use of linked local data.

In Spring 2023 we recruited and trained 15 community researchers in Widnes, UK to co-design and run a project on what feeling well meant for their community. We trained our community researchers in listening skills, methods, and data concepts. Over Summer 2023, they interviewed over 200 of their fellow residents. We then ran three design workshops to analyse their findings and co-design a data and community insights hub with local policymakers and voluntary sector representatives.

Through repeated, comparative semi-structured interviews we tracked the community researchers’ progress. We used thematic analysis to explore the benefits and challenges at each stage of the project: recruitment, training, and field work. Benefits ranged from the practical value of capacity building through the research training to the key role of confidence in how community researchers perceived their own success during the project. Challenges ranged from more esoteric concepts like managing expectations of impact to practical concerns around diversity and interview recruitment. Overall, the project evidenced the value of peer research methodologies in involving public in data science both for the participants but also to wider community.

